# Vitamin D deficiency is associated with increased risk of Alzheimer’s disease and dementia: evidence from meta-analysis

**DOI:** 10.1186/s12937-015-0063-7

**Published:** 2015-08-01

**Authors:** Liang Shen, Hong-Fang Ji

**Affiliations:** Shandong Provincial Research Center for Bioinformatic Engineering and Technique, School of Life Sciences, Shandong University of Technology, Zibo, 255049 P. R. China

## Abstract

**Background:**

In recent years, the associations between vitamin D status and Alzheimer’s disease (AD) and dementia have gained increasing interests. The present meta-analysis was designed to estimate the association between vitamin D deficiency and risk of developing AD and dementia.

**Methods:**

A literature search conducted until February 2015 identified 10 study populations, which were included in the meta-analysis. Pooled risk ratios (RRs) and 95 % confidence interval (CI) were calculated with a random-effect model using Stata software package.

**Results:**

Results of our meta-analysis showed that subjects with deficient vitamin D status (25(OH)D level < 50 nmol/L) were at increased risk of developing AD by 21 % compared with those possessing 25(OH)D level > 50 nmol/L. Similar analysis also found a significantly increased dementia risk in vitamin D deficient subjects. There is no evidence for significant heterogeneity among the included studies.

**Conclusion:**

Available data indicates that lower vitamin D status may be associated with increased risk of developing AD and dementia. More studies are needed to further confirm the associations and to evaluate the beneficial effects of vitamin D supplementation in preventing AD and dementia.

## Introduction

Alzheimer’s disease (AD) is the most common form of dementia in the elderly. With the accelerating population aging process, the prevalence of AD and dementia is estimated to rise steadily [1, 2]. Despite considerable effort has been devoted to the drug discovery for AD, there is no effective agent to combat it at present. Thus, it is urgent to identify specific modifiable risk factors for these disorders.

In recent years, the associations between vitamin D and AD or dementia have attracted growing interests [3–5]. First, accumulating studies indicate that vitamin D deficiency is prevalent in AD and dementia patients [6, 7] and a meta-analysis study supported that AD patients possess lower level of 25-hydroxyvitamin D [25(OH)D] compared with age-matched healthy controls [8]. Second, low 25(OH)D level may be a potential risk factor of developing AD and dementia as supported by recent studies [9, 10]. However, there is a lack of a comprehensive evaluation on whether vitamin D deficiency correlates with high risk of AD and dementia development, which has important implications for the prevention of these disorders. Therefore, the present study was designed to estimate the association between vitamin D deficiency and risk of developing AD and dementia.

## Methods

### Search strategy and study selection

The meta-analysis was performed according to the PRISMA guidelines. With the keywords “Alzheimer’s disease” or “dementia” and “vitamin D” or “25(OH)D”, the literature search was conducted in the MEDLINE database from inception until February 2015. The references lists of retrieved articles were also manually reviewed to identify relevant studies missed by the search strategy. The potentially relevant references were identified for inclusion by reviewing titles and/or abstracts, and/or full text of all citations identified with database searches. Vitamin D deficiency was defined as a serum 25(OH)D, a stable marker of vitamin D status, concentration of ≤ 50 nmol/L, which has been widely used in relative studies as the cut-off point for vitamin D deficiency [11]. For consistency, serum concentrations of 25(OH)D present in nmol/L were converted to ng/mL by using the conversion factor (1 ng/mL = 2.5 nmol/L).

The eligible studies must meet the following inclusion criteria: i) original studies to evaluate the association of vitamin D status and risk of developing AD or dementia; ii) providing the odds ratios (ORs), relative risks (RRs) or hazard ratios (HRs) with 95 % confidence intervals (CI) of developing AD or dementia in vitamin D deficient subjects comparing with subjects with serum 25(OH)D concentration of > 50 nmol/L. The animal experiment, review and mechanistic research studies were excluded. Duplicate articles were excluded in the study. Only references published in English is considered.

### Data extraction and analysis

The collected information from each identified study include: the first author, year of publication, country, average age, OR, and 95 % CI, and adjusted factors. Two reviewers extracted the data from each study independently and finally verified the extracted data. The meta-analysis was performed using the Stata statistical software package, version 12.0 (StataCorp, College Station, Texas, USA). The random effect model was employed during all the analyses. The heterogeneity was assessed through the *I*^2^ statistic.

## Results

### Study characteristics

**Fig. 1:**
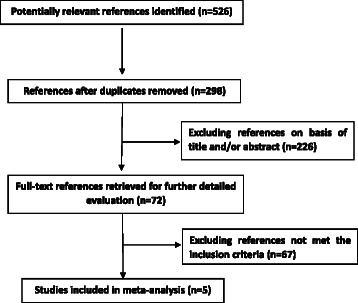
Flowchart of selection of the references for inclusion in meta-analysis

The study selection flowchart was detailed in Fig. 1. Overall, 526 potentially relevant references were initially identified through database search and 298 were obtained for further screen after duplicates removed. Following initial titles and/or abstracts screening 226 references were excluded. After full text assessment of the remained articles five studies, which include 10 study populations, were identified and included in the present analysis [9, 10, 12–14]. For the five eligible studies, two studies are prospective cohort and three are cross-sectional studies. Five eligible studies were conducted in Denmark, USA, UK, France and Germany, respectively. All included studies were published in English. The years of publication ranged from 2010 to 2015.

### Vitamin D deficiency and risk of AD

**Fig. 2:**
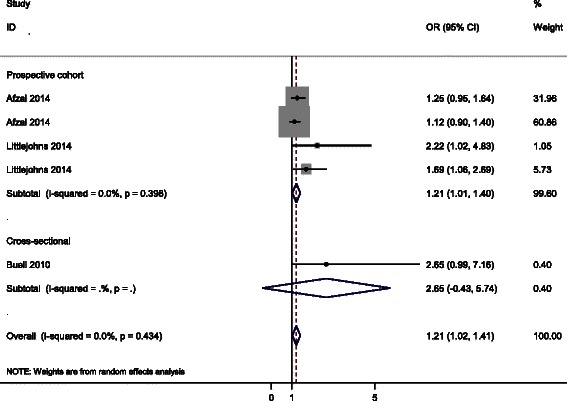
Forest plot of the included studies investigating risk of developing AD in vitamin D deficient subjects. The size of each square is proportional to the study’s weight

Five study populations from three studies were included in the meta-analysis of the association between vitamin D deficiency and risk of developing AD [9, 10, 12]. Main characteristics of the included studies were provided in Table 1. Forest plot of the included studies investigating risk of AD in vitamin D deficient subjects was shown in Fig. 2. Results of the prospective cohort studies showed that vitamin D deficiency was associated with increased risk of AD occurrence compared to the subjects with 25(OH)D level > 50 nmol/L, overall OR = 1.21, 95 % CI 1.01–1.40. The *I*^*2*^ value was 0.0 %, which suggested that there was no evidence for significant heterogeneity. The inclusion of one cross-sectional study negligibly affected the results (Fig. 2).

**Table 1:** Summary characteristics of studies included in the analysis of vitamin D deficiency and risk of AD

References	Year	Country	Study type	Participants	Average age (years)	OR	95 % CI	25(OH)D (nmol/L)	Adjustment
Afzal [10]	2014	Denmark	Prospective cohort	2384	-	1.25	0.95–1.64	<25	Age, sex, month of blood sample, smoking status, body mass index, leisure time and work-related physical activity, alcohol consumption, income level, education, baseline diabetes mellitus, hypertension, cholesterol, high-density lipoprotein cholesterol, and creatinine
Afzal [10]	2014	Denmark	Prospective cohort	4087	-	1.12	0.90–1.4	25–50	Age, sex, month of blood sample, smoking status, body mass index, leisure time and work-related physical activity, alcohol consumption, income level, education, baseline diabetes mellitus, hypertension, cholesterol, high-density lipoprotein cholesterol, and creatinine
Littlejohns [9]	2014	UK	Prospective cohort	1615	73.6 ± 4.5	2.22	1.02–4.83	<25	Age, season of vitamin D collection, education, sex, BMI, smoking, alcohol consumption, and depressive symptoms
Littlejohns [9]	2014	UK	Prospective cohort	1615	73.6 ± 4.5	1.69	1.06–2.69	25–50	Age, season of vitamin D collection, education, sex, BMI, smoking, alcohol consumption, and depressive symptoms
Buell [12]	2010	USA	Cross-sectional	318	73.5 ± 8.1	2.65	0.99–7.16	≤50	Age, race, sex, body mass index, and education, kidney function, multivitamin use, season, diabetes, hypertension, plasma homocysteine, and ApoE allele status

### Vitamin D deficiency and risk of dementia

**Fig. 3:**
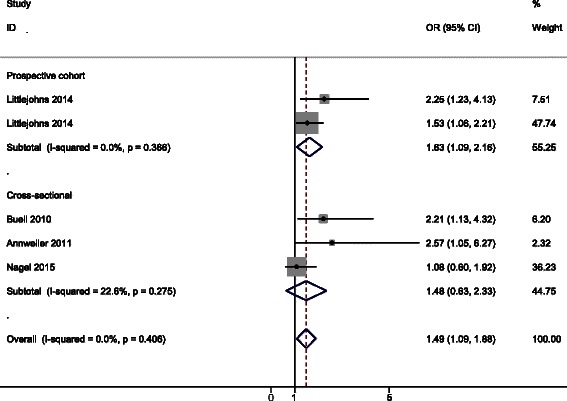
Forest plot of the included studies investigating risk of developing dementia in vitamin D deficient subjects. The size of each square is proportional to the study’s weight

Table 2 provided the summary of the included studies concerning vitamin D deficiency and risk of developing dementia [9, 12–14]. Meta-analysis of the prospective cohort studies showed that the risk of developing dementia was increased by 63 % in comparison with the subjects with 25(OH)D level > 50 nmol/L according to the estimated OR = 1.63, 95 % CI 1.09–2.16 (Fig. 3). Similar results were observed from meta-analysis restricted to prospective cohort studies (OR = 1.48, 95 % CI 0.63–2.33) and the *I*^*2*^ value suggested that there was no evidence for significant heterogeneity among the studies (Fig. 3).

**Table 2:** Summary characteristics of studies included in the analysis of vitamin D deficiency and risk of dementia

References	Year	Country	Study type	Participants	Average age (years)	OR	95 % CI	25(OH)D (nmol/L)	Adjustment
Littlejohns [9]	2014	UK	Prospective cohort	1547	73.6 ± 4.5	2.25	1.23–4.13	<25	Age, season of vitamin D collection, education, sex, BMI, smoking, alcohol consumption, and depressive symptoms
Littlejohns [9]	2014	UK	Prospective cohort	1547	73.6 ± 4.5	1.53	1.06–2.21	25–50	Age, season of vitamin D collection, education, sex, BMI, smoking, alcohol consumption, and depressive symptoms
Buell [12]	2010	USA	Cross-sectional	318	73.5 ± 8.1	2.21	1.13–4.32	≤50	Age, race, sex, body mass index, and education, kidney function, multivitamin use, season, diabetes, hypertension, plasma homocysteine, and ApoE allele status
Annweiler [13]	2011	France	Cross-sectional	288	86.0 ± 0.4	2.57	1.05–6.27	<25	Fully adjusted but without detailed information
Nagel [14]	2015	Germany	Cross-sectional	1373	75.6 ± 6.57	1.08	0.60–1.92	≤50	Adjusted for age, sex, school education, smoking status, season, alcohol consumption, BMI, and history of depression

## Discussion

As a fat-soluble steroid hormone, vitamin D possesses a wide range of health-promoting effects and has potential therapeutic benefits in combating many disorders, including the non-skeletal, age-associated disorders [15–17]. In recent years, the associations between vitamin D and AD or dementia have attracted increasing attentions [3–5, 18–21]. The meta-analysis by Etgen et al. found that participants with low vitamin D status showed an increased risk of cognitive impairment compared with normal vitamin D status [22]. Similarly, Balion et al. found that AD patients had a lower vitamin D concentration compared with controls and participants with higher vitamin D concentrations had a higher average Mini-Mental State Examination score through meta-analyses [23]. The present study was designed to further explore whether low vitamin D status predicts increased incidence of AD and dementia. The data showed that subjects with deficient vitamin D (serum 25(OH)D level ≤ 50 nmol/L) were at higher risk for the development of AD and dementia in comparison with those with serum 25(OH)D level > 50 nmol/L. Findings of this meta-analysis are indirectly supported by a recent study covering 5010 subjects free of dementia at baseline indicating that higher vitamin D concentration is associated with lower dementia risk after a 17-year follow up [20].

Attempts have been made to explore the effects of vitamin D supplementation in preventing AD or dementia in recent years, while the results are inconsistent [24–26]. In a prospectively followed cohort study of 498 older women aged 75 years and older, it was found that higher vitamin D dietary intake can lower the risk of developing AD after a 7 years follow-up [24]. In comparison, in a randomized double-blind placebo-controlled trial recruiting 4143 women aged 65 and older without probable dementia at baseline, no association between vitamin D combined with calcium carbonate treatment supplement and incident cognitive impairment was observed [26]. Thus, further studies recruiting a large number of participants by considering the gender and ethnic differences and with different vitamin D dosages are encouraged to evaluate the efficacy of vitamin D supplementations in preventing AD and dementia.

Some limitations in the present meta-analysis need to be considered. First, the number of eligible studies is relatively small. Second, men and women have different AD incidence overall [27, 28], while there are not enough data to perform gender subgroup analysis to explore the effect of gender on the association between vitamin D deficiency and risk of developing AD. Third, the available data cannot permit us to exclude the possibility that the associations between vitamin D and AD or dementia are a result of disease development rather than being causal.

## Conclusions

In summary, available data shows that vitamin D deficiency may be associated with increased risk of developing AD and dementia. There is a strong need to further confirm the associations by more prospective cohort studies. In addition, in view of the safe and cost-effective interventions to improve vitamin D status, the potential beneficial effects of vitamin D supplementation in preventing AD and dementia should be paid attention to and assessed by the neurologists and geriatricians.
